# Congenital diaphragamatic hernia associated with aortic coarctation: a case report

**DOI:** 10.1186/1757-1626-1-378

**Published:** 2008-12-08

**Authors:** Manabu Okawada, Toshihiro Yanai, Atsuyuki Yamataka, Tadaharu Okazaki, Hiroyuki Kobayashi, Shiori Kawasaki, Geoffrey J Lane, Takeshi Miyano

**Affiliations:** 1 Department of Pediatric General and Urogenital Surgery, Juntendo University School of Medicine, 2-1-1 Hongo, Bunkyo-ku, Tokyo 113-8421, Japan; 2 Cardiovascular Surgery, Juntendo University School of Medicine, 2-1-1 Hongo, Bunkyo-ku, Tokyo 113-8421, Japan

## Abstract

Congenital diaphragmatic hernia associated with cardiac anomalies is a major therapeutic challenge. We report a case of Congenital diaphragmatic hernia associated with coarctation of the aorta.

## Introduction

Up to 40% of patients with congenital diaphragmatic hernia (CDH) have associated anomalies. Of those, cardiac anomalies, the most common, are present in 63% [1]. Treatment of CDH with cardiac anomalies is a major challenge for pediatric and cardiovascular surgeons. We report a case of CDH with aortic coarctation that was treated successfully.

## Case presentation

**Figure 1:**
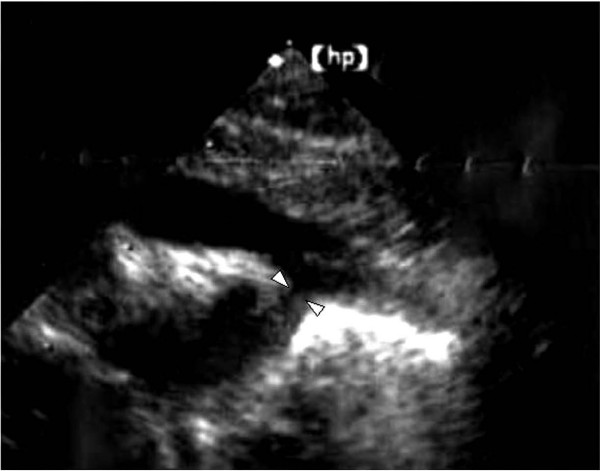
Echocardiography demonstrated a 1.5 mm diameter preductal coarctation of the aorta (CoA) (arrows).

A 2.6 kg female was delivered by planned cesarean section at 38 weeks gestation with known CDH diagnosed prenatally. She was immediately intubated and started on ventilatory support with high frequency oscillatory ventilation (HFOV). Echocardiography (EC) demonstrated a 1.5 mm diameter preductal coarctation of the aorta (CoA), a large (6 mm) patent ductus arteriosus (PDA) and continuous right-to-left shunting pattern, and a 3 mm membranous ventricular septal defect (VSD), which were not suspected prenatally (Fig. 1). Tricuspid valve reflux (TR) was mild/moderate and left pulmonary artery (lPA) blood flow was poor. Hemodyamics examined by EC were typical of coarctation complex. Prostaglandin E1 (PGE1) at a dosage of 5 μg/kg/min was administrated to maintain the circulation because of the CoA. Inhaled nitric oxide (NO) at 10 ppm was used to diminish persistent pulmonary hypertension (PPH). Cardiovascular status and post-ductal oxygen saturation could be maintained without extracorporeal membrane oxygenation (ECMO) for 24 hours after birth allowing the CDH to be repaired by direct primary closure. PDA patency and post-ductal circulation were maintained postoperatively. Seven days after CDH repair, the PDA was ligated and the CoA corrected by end-to-end anastomosis. However, 3 days after correction of the CoA, there was sudden deterioration in renal function and a stricture of the anastomotic site of the aorta was confirmed by EC. It was 1.8 mm and the pressure gradient was 17 mmHg. The Ejection Fraction was 65.8% and cardiac function was stable, allowing re-anastomosis to be performed on the same day. Although transient acute renal failure developed, postoperative recovery was satisfactory. Now 3 years old, she remains well, without any need for oxygen or medications.

## Discussion

The association of CDH with cardiovascular malformations has been well described, but therapies have changed over the years. Cohen et al reported on the influence of congenital heart disease on the survival of children with CDH and only 5 of 16 patients survived [2]. CoA often coexists as a component of left ventricular hypoplasia or small left ventricle [3-6], which is almost universally fatal and is now increasingly recognized as the most commonly associated cardiac malformation associated with CDH [2]. However, in cases of CDH with isolated CoA, the ultimate outcome is related to the extent of pulmonary hypoplasia and persistent pulmonary hypertension not the CoA, which is a correctable cardiac malformation. In the present case, cardiovascular and respiratory status were maintained using a combination of HFOV, NO, vasoactive agents such as PGE1 and catecholamines without ECMO. We guess that our good outcome depended on mild pulmonary hypoplasia and persistent pulmonary hypertension, although the tight preductal CoA and VSD in combination with a large PDA resulted in right to left shunting for post CoA aortic flow.

ECMO is generally indicated in severe cases of CDH. Interestingly, the accuracy of prenatal diagnosis of CDH is not matched by the accuracy of fetal EC for predicting associated cardiac malformations. The diagnosis of cardiac malformation is often made at failure of weaning from ECMO or at autopsy, despite apparently normal EC at birth [7,8]. Prenatal and/or postnatal diagnosis of cardiac malformation by EC is essential for appropriate management of CDH with cardiac malformations including the decision whether or not to use ECMO.

In the present case, correction of CoA was performed seven days after CDH repair. The timing of surgery was determined by improvement in PPH, such as no need for NO, disappearance of TR, and increased lPA blood flow. EC is also indispensable for determining the proper timing of cardiovascular surgery.

Successful management of CDH associated with cardiac malformations largely depends on the severity of pulmonary hypoplasia and persistent pulmonary hypertension, and the maintainance of cardiovascular function. The early and appropriate diagnosis of cardiac anomalies and the appropriate timing of surgical intervention using EC improve the outcome of CDH with cardiac malformations.

## Abbreviations

CDH: Congenital Diaphragmatic Hernia; HFOV: High Frequency Oscillatory Ventilation; EC: Echocardiography; CoA: Coarctation of the Aorta; PDA: Patent Ductus Arteriosus; VSD: Ventricular Septal Defect; TR: Tricuspid valve Reflux; lPA: left Pulmonary Artery; PGE1: Prostaglandin E1; NO: Nitric Oxide; PPH: Persistent Pulmonary Hypertension; ECMO: Extracorporeal Membrane Oxygenation

## Consent

Written informed consent was obtained from the patient for publication of this case report and accompanying images. A copy of the written consent is available for review by the Editor-in-Chief of this journal.

## Competing interests

The authors declare that they have no competing interests.

## Authors' contributions

MO did the literature search and wrote the case report and also obtained written consent. AY, TO conceived the study and helped to draft the manuscript. TY, HK, SK, GJL and TM prepared the manuscript and helped in the literature search. All authors had gone through the final manuscript and approved it.
